# A Scoping Systematic Review of Cannabis Use in Endometriosis

**DOI:** 10.1111/ajo.70081

**Published:** 2025-12-09

**Authors:** Kindha McLaren, Simon Erridge, Mikael H. Sodergren

**Affiliations:** ^1^ Medical Cannabis Research Group Imperial College London London UK; ^2^ Curaleaf Clinic London UK

**Keywords:** cannabidiol, cannabis, chronic pain, endometriosis, tetrahydrocannabinol

## Abstract

**Background:**

Endometriosis, affecting 6%–10% of reproductive‐age women, causes chronic pelvic pain, dysmenorrhea, and infertility. Current treatments have limitations and consequently there is rising interest in effects of cannabis on pain and inflammation associated with endometriosis.

**Aims:**

This scoping review primarily aimed to characterise the effects of cannabis on endometriosis‐associated pain and detail the reported adverse events.

**Materials and Methods:**

A search was collected on PubMed, MEDLINE (Ovid), and EMBASE (Ovid) databases on 16th January 2024. Studies were included if they were in‐human or clinical studies evaluating the effects of cannabis in endometriosis in non‐pregnant adults.

**Results:**

Thirteen studies, including 4 ongoing studies, were included. All nine completed studies, with 1,787 participants, were cross‐sectional. Pain (57.3%–95.5%) was the most common indication for cannabis use, followed by sleep and gastrointestinal distress (15.2%–78.5%). Cannabis was most commonly inhaled (51.6%–80.3%) or ingested (25%–76.9%). Eight (61.5%) studies asked about participants' perception of the efficacy of cannabis. These utilised a range of methods preventing pooling of results. However, most reported improvement in at least a proportion of their studied population. Adverse events were reported by 10.2% to 52.0% of patients, with the most common being “feeling high” (euphoria) and a dry mouth.

**Conclusions:**

Several observational studies have reported that cannabis helped to reduce endometriosis‐associated pain. However, there is a paucity of high‐quality prospective longitudinal data and randomised controlled trials to evaluate the safety profile an efficacy of medical cannabis in endometriosis‐associated pain. These provide support, alongside existing pre‐clinical data, for the importance of further assessment in randomised controlled trials.

## 1 Introduction

Endometriosis, affecting 6%–10% of reproductive‐age women, is a chronic inflammatory disease characterised by endometrial tissue growth outside the uterus [1, 2]. Symptoms include dysmenorrhoea, pelvic pain, and infertility [1, 2]. Pain is a central feature, significantly impacting quality of life, with over 50% of patients experiencing severe pelvic pain since adolescence [2, 3]. This pain often co‐occurs with other chronic pain conditions like fibromyalgia, and contributes to mental health issues, such as depression and anxiety [1, 4].

The aetiology of endometriosis remains unknown. Yet, a leading theory is retrograde menstruation, where menstrual tissue flows back through the fallopian tubes [1]. This process, alongside neuroangiogenesis, contributes to chronic inflammation and activation of pain pathways. However, this theory does not fully explain the complexity of the disease, as retrograde menstruation is more common than endometriosis itself [1, 4].

Management of endometriosis‐associated pain is challenging, as it encompasses nociceptive, neuropathic, and nociplastic pain [1]. Current treatments include surgical interventions such as excision and ablation, which can provide significant pain relief [4]. Yet surgery often only provides temporary relief, with symptoms recurring in up to three‐quarters of women within 2 years [5]. A study found that over a decade, 51% of women undergoing laparoscopic surgery required repeat procedures [6]. Hormonal therapies, including combined oral contraceptives, progestogens, and gonadotrophin‐releasing hormone analogues are commonly used to manage endometriosis and reduce pain significantly. However, pain persists in up to 60% of treated patients, with 20% experiencing no relief [7]. Furthermore, treatment efficacy is limited to the duration of use, and symptoms recur in up to 74% of patients upon discontinuation [4, 7]. While non‐steroidal anti‐inflammatory drugs (NSAIDs), such as naproxen and ibuprofen may effectively treat primary dysmenorrhea, their impact on endometriosis‐associated pain is unclear [8]. Despite frequent opioid prescribing, clinical evidence supporting their efficacy in this context is also lacking [9, 10].

Due to the prevalence of endometriosis and the challenges in addressing associated pain, there is a need to identify novel therapies. The endocannabinoid system is involved in the modulation of pain and inflammatory activity [11]. Endogenous ligands bind to G protein‐coupled receptors cannabinoid receptors type 1 (CB1R) and type 2 (CB2R). Peripherally, in the nociceptive terminals and the dorsal root ganglion, CB1R inhibits neurotransmitter release and pain transmission [12]. In medullary areas, CB1R activates the descending inhibitory pathway via inhibition of GABA release [12]. CB2R regulates the immune response in the spinal cord, sensitising neurones during chronic pain. In immune cells and keratinocytes, it diminishes the release of pronociceptive agents [12].

Cannabis contains over 140 phytocannabinoids, including the two principal components—(−)‐*trans*‐Δ^9^‐tetrahydrocannabinol (THC) and cannabidiol (CBD). Both act on CB1R and CB2R—THC is a partial agonist whilst CBD is a negative allosteric modulator [13, 14]. Cannabinoids also act on transient receptor potential vanilloid subtype 1 (TRPV1), which mediates thermal hyperalgesia [15]. TRPV1 also modulates neuropathic pain through interaction with CB2R. Endogenous cannabinoids activate TRPV1 and cannabinoid receptors, inhibiting neuropeptide release and blocking inflammatory hyperalgesia [15].

Current evidence for the cannabis‐based management of endometriosis‐associated pain is of low quality. A systematic review of randomised controlled trials (RCTs) for cannabinoids, cannabis, and cannabis‐based medicine in pain management found that evidence was inconclusive due to high and/or uncertain levels of bias and little confidence in estimates of effects [16]. Another review analysed RCTs comparing cannabinoids against placebo in patients with any type of pain [17]. Of the 65 included, 59 trials, and all outcome results, had high risks of bias. This is partly due to small sample sizes, implicit bias, and limited follow up [16, 17, 18].

There is heterogeneity in the medicines studied as well as the conditions assessed, with few reviews including substantial evidence on the role of cannabis in endometriosis. A meta‐analysis by Wang and colleagues concluded with moderate to high certainty that non‐inhaled medical cannabis use in chronic pain results in a small to very small improvement in pain relief [18]. However, it only included one study concerning endometriosis, examining the efficacy of palmitoylethanolamide (PEA), a fatty acid amide which is not derived from the cannabis plant and does not bind to cannabinoid receptors. Many studies are also affected by short follow up, meaning that the characteristics of long‐term cannabis use in chronic pain are unknown [17, 18]. There is a need to review the current clinical landscape around the medical effects and safety of cannabis for patients with endometriosis. This scoping systematic review aims to analyse available evidence from clinical research to evaluate the efficacy and safety of cannabis in managing pain, quality of life, and adverse effects in adult patients with endometriosis.

## 2 Methods

A scoping systematic review was performed on in‐human literature on the effects of cannabis in adults with endometriosis. The review was conducted and reported in accordance with the Preferred Reporting Items for Systematic reviews and Meta‐Analysis extension for Scoping Reviews checklist [19]. As a systematic review of previously published literature this study is exempt from ethical review.

### 2.1 Research Question

Due to the diversity of reported use of cannabis in individuals with endometriosis, the primary objective of this review was to explore the clinical literature reporting outcomes in individuals taking cannabis with and without medical supervision. Secondary objectives included evaluation of product and patient factors in individuals taking cannabis for endometriosis‐associated chronic pain.

All pain‐specific and other reported health effects reported by individuals consuming cannabis in each study were noted, including adverse effects. Considering the paucity of data on outcomes in this patient cohort, no limitations were set on the sources or uses of cannabis. Therefore, consumers of illegal and legal adult‐use cannabis products were included alongside those prescribed cannabis‐based medicinal products.

### 2.2 Search Strategy

A comprehensive search was conducted utilising MEDLINE (Ovid), PubMed, EMBASE (Ovid) (Tables S1–S3). The searches were all conducted on 16 January 2024. MEDLINE was updated from 1946 to 12 January 2024. PubMed was updated to 15 January 2024. EMBASE was updated from 1947 to 12 January 2024 (Supporting Information S1).

The following combination of search terms were used: “Endometriosis” OR “Endometrio$” AND “Cannabis” OR “Hemp” OR “Marijuana” OR “Ganja” OR “Hashish” OR “Marihuana” OR “Bhang” OR “Cannabinoid” OR “Dronabinol” OR “Marinol” OR “Nabilone” OR “Cesamet” OR “HU 211” OR “Dexanabinol” OR “Nabiximols” OR “Sativex” OR “Dronabinol” OR “Tetrahydrocannabinol” OR “Cannabidiol” OR “Epidiolex” OR “Epidyolex” OR “Cannabinol”. The search was not limited to English.

Studies were included in this scoping review if they were in‐human or clinical studies evaluating the effects of medical cannabis in endometriosis in non‐pregnant people aged 18 and over. Studies were excluded if they were pre‐clinical studies or non‐original research. Studies included were RCTs, cross‐sectional studies, cohort studies and case series.

Two authors (K.M. and S.E.) independently reviewed the titles and abstracts of included articles. Studies included after this stage underwent a full text review. Discrepancies were resolved by a senior author (M.H.S).

### 2.3 Assessment of Quality

The quality of each study which met inclusion criteria was assessed using the Newcastle‐Ottawa Scale [20]. This comprises a score ranging from 0 to 9, with points assigned according to participant selection, comparability of studied groups, and outcome or exposure.

### 2.4 Data Extraction

Data extraction was performed by two authors (K.M. and S.E.). The following information was extracted from each of the eligible studies (where the relevant information was provided): study design, population characteristics (country, age, severity of diagnosis), reported efficacy of cannabis, proportion of patients reporting adverse events, proportion of patients able to reduce their prescribed medication following cannabis use, frequency of cannabis usage, mode of cannabis use, and type of cannabis used.

## 3 Results

**FIGURE 1:**
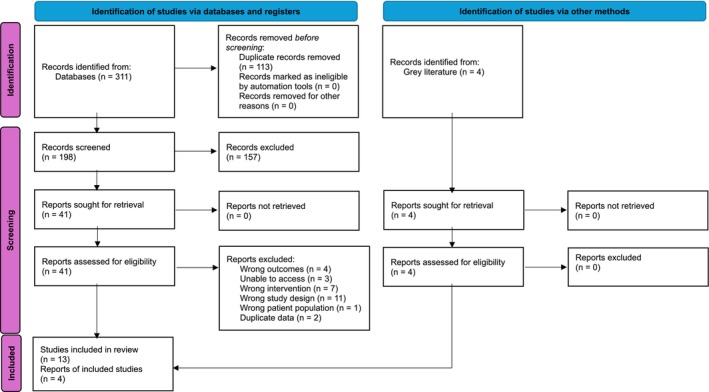
PRISMA flowchart [21].

Three hundred and fifteen results were yielded from the database search, with an additional 4 studies coming from ClinicalTrials.gov (Figure 1). From these, 121 duplicates were identified and removed, leaving 198 studies. A further 157 studies were excluded, 41 underwent full text review, and 13 met the inclusion criteria (Table 1). Four studies were ongoing at the time of submission, as detailed in Table 2.

**TABLE 1:** Background and design of included studies.

Author name	Year	Location	Newcastle‐Ottawa score	Number of cannabis users	Mean age (Years)	Investigation	Diagnosis of endometriosis	Severity of endometriosis	Method of cannabis usage	Frequency of cannabis usage
Armour et al. [22]	2019	Australia	5	48	31 ± NR	Online questionnaire	Laparoscopic diagnosis	NR	NR	NR
Armour et al. [23]	2021	New Zealand	3	213	44.1 ± 13.9	Online questionnaire	Self‐reported	NR	65.7% used a cooked recipe 25.8% used oil‐filled capsules 37.1% consumed as oil or tincture 80.3% smoked as a cannabis cigarette 63.4% used a pipe 68.1% used a water pipe (bong) 40.4% inhaled through a vaporiser 43.7% used topical applications	70% used daily 20.9% used weekly
Armour et al. [24]	2022	46 countries	5	846	30.7 ± 7.1	Online questionnaire of cannabis use in the past 12 weeks during the COVID‐19 pandemic	82.2% surgically diagnosed 7.6% US/MRI 7.5% diagnosed by symptoms 1.7% diagnosed by unspecified method	Mean pain score on EHP‐30 of 68.1 ± 15.3	12.4% used a cooked recipe 12.6% used oil‐filled capsules 24.4% smoked as a cannabis cigarette 13.6% used a pipe 13.6% used a water pipe (bong) 5.6% used topical applications	61.2% used at least once a day 24.7% used at least once a week
Carrubba et al. [25]	2021	United States	6	26	NR	Cross‐sectional survey	Diagnosis of pelvic and perineal pain, dyspareunia, or endometriosis based on ICD‐10	NR	76.9% ingested CBD or THC 61.5% inhaled CBD or THC 30.8% used topical CBD or THC	48% used daily 24% used once weekly 12% used once monthly
Reinert et al. [26]	2019	United States	4	135	30.05 ± 6.7	Survey	Diagnosis of endometriosis based on ICD‐10	NR	NR	NR
Russo et al. [27]	2022	United States	4	3	NR	Online questionnaire	NR	NR	NR	NR
Sinclair et al. [28]	2021	Canada	4	252	35	Analysis of cannabis tracking mobile application data	Self‐reported	NR	32.3% ingested cannabis 67.4% inhaled cannabis (of these, 40.6% vaporised)	NR
Sinclair et al. [29]	2022	Australia & New Zealand	3	237	NR	Online questionnaire	Medical diagnosis	NR	NR	NR
Sinclair et al. [30]	2023	Australia & New Zealand	5	27	NR	Virtual focus group	91.9% surgically diagnosed 5.4% diagnosed based on symptoms 3.7% diagnosed via ultrasound or MRI	NR	NR	NR

Abbreviations: CBD, cannabidiol; EHP‐30, endometriosis health profile‐30; ICD‐10, international classification of disease 10th edition; MRI, magnetic resonance imaging; NR, not recorded; THC, –(−)‐trans‐Δ^9^‐tetrahydrocannabinol; US, ultrasound.

**TABLE 2:** Ongoing studies identified from ClinicalTrials.gov.

Clinical trial ID	Sponsor	Location	Study Type	Estimated Completion Date	Demographic	Estimated Enrolment	Intervention	Primary Outcome
NCT05670353	University of Sao Paulo	Brazil	Randomised double‐blind placebo‐controlled trial	20/08/2024	Adult women with refractory symptoms of chronic pelvic pain secondary to surgically treated endometriosis	102	Oral CBD 10 mg (up titrated until 150 mg or adverse effects) daily for 9 weeks	Proportion of women with ≥ 30%, and ≥ 50% change in pain intensity (measured through visual analogue scale)
NCT04527003	Milton S. Hershey Medical Center	United States	Randomised double‐blind placebo‐controlled trial	01/12/2024	Women aged 18 to 45 years with a surgical diagnosis of endometriosis and associated moderate to severe endometriosis related pain (> 3 on a visual analogue scale)	36	Daily administration of norethindrone acetate 5 mg + either sublingual CBD 10 mg or sublingual CBD 20 mg	Total pain score reported daily using a 0–100 visual analogue scale
NCT04565470	Benno Rehberg‐Klug	Switzerland	Observational questionnaire	01/06/2024	Women aged ≥ 18 years consulting at a specialised endometriosis centre	150	N/A	Frequency of use of any self‐management strategy during the last 6 months
NCT03875261	David Garcia Cinca	Spain	Single‐arm interventional study	15/07/2019	Women aged 18 to 40 years with a diagnosis of deep endometriosis confirmed on imaging and pain of ≥ 4 on a numerical visual scale of 11 levels	10	1–12 sprays of nabiximols (2.7 mg THC and 2.5 mg CBD per spray)	Pressure threshold in hypogastrium that induces pain measured in kPa

Abbreviations: CBD, cannabidiol; kPa, kilopascals; mg, milligrams; N/A, not applicable; THC, (−)‐trans‐Δ^9^‐tetrahydrocannabinol.

Five (38.0%) studies involved participants from the United States, four (30.7%) from Australia, four (30.7%) from New Zealand, and one (7.7%) each from Canada, Switzerland, and Brazil. All nine (69.0%) completed studies were cross‐sectional. Of nine completed studies, seven (77.8%) utilised cross‐sectional online surveys, one (11.1%) used a combination of surveys and focus groups, and one (11.1%) analysed data from a cannabis usage tracking app. Two (22.2%) studies only examined patients with endometriosis using cannabis at the point of survey, with an additional study (11.1%) examining cannabis users of which a small subset had endometriosis. In seven (77.8%) studies, participants were recruited via social media. One (11.1%) study recruited from a clinic mailing list, and one (11.1%) study recruited eligible candidates from outpatient clinic appointments. Most studies could not calculate response rates due to the methods of recruitment. Of the two (22.2%) that did, response rates were 47.1% and 75.2% [4, 5]. Participant sample sizes ranged from 3 to 846.

The median Newcastle‐Ottawa score was 4 (range: 3–6). As all studies relied on self‐report to determine the effects of cannabis, they may have suffered from recall bias, selection bias, sampling bias, and nonresponse bias.

Eight (61.5%) studies provided information on confirmation of diagnosis with endometriosis, although in six (46.2%) studies diagnoses were self‐reported. Carrubba et al. [25] reported that 40.5% of patients had seen > 5 doctors before receiving a diagnosis, and 56.7% had seen 2–5. Stage of endometriosis at last surgery was also recorded, from Stage 2 (20.6%), Stage 3 (32.6%), and Stage 4 (29.4%). No other studies reported on the stage of endometriosis.

Five (38.5%) studies explored patients' reasons for using cannabis. The most common indications were pain (57.3% to 95.5%), sleep improvement (95.5% of users in Armour et al. [24]), and gastrointestinal distress (15.2%–78.5%). Other reasons given included increasing ability to cope [3], cramps [8], and mood [8]. Almost half (49.8%) of consumers in Sinclair et al. [30] first started using cannabis after noticing symptom reduction on recreational use. Others within the study had received recommendations from endometriosis support groups (39.2%), friends with endometriosis (30.0%), or started due to difficulty finding access to medical expertise on endometriosis (24.5%). Themes highlighted within a focus group in Sinclair et al. [28] included users reporting that they turned to cannabis due to the failure of orthodox medical treatments to adequately relieve their symptoms. Others within the group noted that they did not experience a “high” when using cannabis for therapeutic purposes.

Some patients also used cannabis recreationally, with 55% of cannabis users in Armour et al. [23] and 25.9% in Sinclair et al. [28] reporting that they used cannabis purely to manage their symptoms.

Eight (61.5%) studies asked about participants' perception of the efficacy of cannabis (Table 3). Pain relief was the most reported reason for use, ranging from 57.3% to 95.5%. Four (30.8%) studies reported on the method of administration, three (23.1%) on the frequency of use, and two (15.4%) on the doses of cannabis. Table 1 shows that cannabis was most commonly inhaled or ingested, with ranges from 51.6% to 80.3% and 25% to 76.9%, respectively. Most women used cannabis at least once daily (ranging from 48% to 70%). Topical methods were reported to be more effective compared to inhaled in Sinclair et al. [29], with efficacy ratings relative to inhalation of 13.6 (6.1–21.2), though a smaller number of sessions were analysed (0.3% of total sessions). In the same study, oral methods of administration were reported as less effective when compared to inhalation with an efficacy rating of −2.1 (−3.2 to −1.0). Of cannabis users in Carrubba et al. [25], 24.0% used CBD with dosages ranging from 1 to 2000 mg per day and 12.0% used THC with doses between 1 and 70 mg per day. The majority (60.0%) used a combination of THC and CBD [25]. There was an increase in improvements in pain, cramping, and muscle spasms in those using a combination of CBD and THC (*n* = 15, 100%) when compared with CBD (*n* = 3, 50%) or THC alone (*n* = 2, 66.7%) (*p* = 0.01) [25]. THC users were found to be significantly more likely to inhale cannabis, but no difference was found in frequency of use between CBD, THC, and combination users [25].

**TABLE 3:** Outcomes of included studies.

Author name	*N* using cannabis at the time (%)	Effectiveness	Adverse effects	Reduction of other medicines
Armour et al. (2019)	48 (9.9%)	Self‐reported effectiveness of 7.6 ± 2.0 on a 0–10 scale	10.2% reported adverse events including drowsiness, increased anxiety, tachycardia	56.3% reported reduction of endometriosis‐related medication by > 50% due to cannabis 27.1% reported reduction of endometriosis‐related medication by 25%–50% due to cannabis
Armour et al. (2021)	213 (100%)	23.0% reported that only cannabis gave them relief from their condition 39.4% reported that cannabis works better than other medicines 95.5% used cannabis for pain relief 91.8% of these patients reported improvement in their pain due to cannabis	34.7% reported no adverse effects 39.9% reported that cannabis had somewhat or much better adverse effects than their usual medication	81.4% reported that cannabis had changed their prescribed medication use 45.5% reported reduction of endometriosis‐related medication by > 50% due to cannabis 40% of medications which were completely stopped were opioids 19.2% reported reduction of endometriosis‐related medication by < 50% due to cannabis
Armour et al. (2022)	846 (50.4%)	Comparing EHP‐30 scores between those using cannabis in the last 12 weeks with those who had not, those using cannabis had worse symptoms and health‐related quality of life. These changes were likely clinically significant. 11.4% of people who previously used cannabis before the start of the COVID‐19 reported that they stopped as they found it to be ineffective	28% of those using cannabis reported at least one side effect 75% reported feeling “high” 67% reported dry mouth 35% reported feeling mild anxiety or paranoia < 1% reported medically diagnosed cannabis hyperemesis syndrome	NR
Carrubba et al. (2021)	26 (23.0%)	84.0% reported improved pain, cramping, and muscle spasms 72.0% reported improved irritability, depression, and anxiety 68.0% reported improved sleeplessness and insomnia	52.0% reported central nervous system adverse events 36.0% reported gastrointestinal adverse events 24.0% reported feeling “high” 16.0% reported no side effects	38.5% reported decreased number of clinical visits to provider (including emergency department and outpatient visits) 30.8% reported reduction in use of opioid medications
Reinert et al. (2019)	135 (37.1%)	71.1% reported cannabis is very or moderately effective 57.3% reported CBD is very or moderately effective	NR	NR
Russo et al. (2022)	3 (2.4%)	Self‐reported effectiveness of 1.67 on a 1–7 scale 66.7% reported cannabigerol‐predominant cannabis is more effective than conventional medicine	NR	NR
Sinclair et al. (2021)	252 (100%)	Cannabis mean efficacy rating = 32.0 (95% CI: 31.3–32.7) 57.3% reported use of cannabis for pain relief 29.1% reported use of cannabis for relief from gastrointestinal distress 13.5% used cannabis for mood	NR	NR
Sinclair et al. (2022)	237 (100%)	72.2% reported use of cannabis for pain relief	NR	> 50% reduction was observed in nonopioid analgesia (63.1%), opioid analgesia (66.1%), hormonal therapies (27.5%), antineuropathics (61.7%), antidepressants (28.2%) and antianxiety medications (47.9%) Of medications completely stopped, 31.1% were opioids. 51.1% of medications completely stopped were antineuropathics.
Sinclair et al. (2023)	27 (73%)	NR	NR	NR

Abbreviations: CBD, cannabidiol; COVID‐19, coronavirus disease‐19; EHP‐30, endometriosis health profile‐30; NR, not recorded.

In Sinclair et al. [29], inhalation was the method with the highest median dose of 9 mg/mL or puff (smoked or vapourised) (IQR: 5, 11, *n* = 10,914) and the lowest doses were seen in those using oral methods for treating pain and mood, at a median dose of 1 mg/mL, capsule or piece (IQR: 0.5–2, *n* = 5220). Topical administration (median dose 2 mg/mL, IQR: 1.5–20, *n administrations* = 53) was the most efficacious ingestion method, with a rating of 13.6 (95% CI 6.1–21.2, *p* = 0.0004) [29]. Additionally, no difference in efficacy between inhalation and ingestion was found when treating pelvic pain [29]. Sinclair et al. [29] also reported that relief of gastrointestinal distress was more effective compared to pain, with a self‐reported efficacy of 9.0 (8.2–9.9) on a scale of 0–10 of pain severity. Ingested forms had a higher efficacy than inhalation for treating mood or gastrointestinal issues with a rating of 5.2 (95% CI 3.0–7.4, *p* < 0.0001) and 7.8 (95% CI 6.3–9.4, *p* < 0.0001) respectively [29]. In this study, self‐rated efficacy of cannabis increased by 1.7 (95% CI 1.2–2.2, *p* < 0.0001) for every ten‐fold increase in THC when examining the THC/CBD ratio. For ingestion, there was a reduced self‐rated efficacy of cannabis of 4.8 (95% CI 5.6–3.89) for every ten‐fold increase in THC compared to CBD [29]. It was found that age was significantly associated with an increased self‐rated efficacy of cannabis of 0.3 (95% CI 0.2–0.3, *p* < 0.0001) for each year increase in age [29].

Four (30.8%) studies compared the effect of cannabis use on conventional prescribed medications. Sinclair et al. [30] reported that 58.2% of cannabis users had started due to intolerable side effects from their conventional medication, with 23.0% participants in Armour et al. [24] stating that only cannabis provided relief from their condition. Two thirds (66.7%) of patients in Russo et al. [27] reported that cannabigerol‐predominant cannabis is more effective than conventional medicine, with the remainder stating that it was equally as effective.

Of the four (30.8%) studies that reported on the impact of cannabis use on use of conventional medications, all found that most patients were able to reduce their conventional medications (Table 3). Opioids were reduced or completely stopped in three out of four studies. Sinclair et al. [30] found that 51% were able to completely stop antineuropathic analgesia and 31.1% completely stopped opioids. Within the sample, 79.7% were on non‐opioid medications at the time and 54.0% were on opioids. Other drugs that were completely stopped include anti‐anxiety medications (27.1%), anti‐depressants (21.9%), hormonal treatments (20.6%), and non‐opioid analgesics (17.2%) [30]. Non‐opioid analgesics and opioids were reduced by > 50% in 45.9% and 35% of users, respectively. In Armour et al. [24] some medications were completely stopped, including opioids (40.0%), antidepressants (16.0%), NSAIDs (17.0%), and benzodiazepines (15.0%). In 16.0% and 33.0% of patients, NSAIDs and opioids were reduced by ≥ 50% respectively. Paracetamol was reduced by < 50% in 41.0% of patients.

Four (30.8%) studies reported on the adverse effects of cannabis use, with ranges of 10.2% to 52.0% of respondents reporting at least one side effect (Table 3). The most common adverse effects were “feeling high” (euphoria), a dry mouth, and central nervous system symptoms such as memory problems, confusion, and sleepiness. Armour et al. [23] found that 22.8% of cannabis users had stopped using cannabis due to unpleasant experiences/unwanted side effects.

Three (23.1%) studies compared the adverse effects between cannabis and conventional medicines (Table 3). In Armour et al. [24], 34.7% of cannabis users reported no adverse effects versus 2.3% of those using conventional medicines. 3.3% reported no difference in adverse effects, with a further 2.3% reporting that cannabis has somewhat or much worse adverse effects compared to usual meds. Carrubba et al. [25] found that euphoria was more likely when using THC (100%, *n* = 3) than with CBD (0%, *n* = 6) (*p* = 0.01).

Four (30.8%) studies explored barriers to using medical cannabis. Carrubba et al. [25] stated that despite medical cannabis being legal, only 19.0% of users utilised their state's cannabis programme. Armour et al. [23] found a positive association between the legality of access to cannabis in a given country and cannabis use in the previous 12 weeks (for any reason) [χ^2^ (1, *n* = 1634) = 88.39, *p* < 0.0001], as well as legality and intention to tell a healthcare professional about their cannabis use [χ^2^ (1, *n* = 1182) = 132.2, *p* < 0.0001].

Sinclair et al. [28] explored the difficulty in accessing affordable quality‐assured medical cannabis. Patients described being apprehensive about using money for cannabis that would otherwise have gone towards conventional medicines and healthcare. Even in patients that could afford it, they were still sometimes unwilling, with one patient reporting that they would not pay the amount of money being charged for their prescribed CBD [28]. Patients also highlighted the lack of variety in available dosages, meaning they were unable to secure the suitable product for them. However, illicit cannabis was often found to have no consistency in efficacy or quality, raising concerns around safety [28]. 31.3% of participants in Sinclair et al. [30] said they had no intention to inform their doctor of their cannabis use due to fear of legal repercussions. Patients were also uncomfortable using cannabis in regions where detection of THC in the bloodstream may constitutes an offence whilst driving or in other areas of life, such as Australia [28].

Issues of the stigma surrounding cannabis use were discussed in two (15.4%) studies. Patients in Sinclair et al. [28] discussed concerns about the stigma surrounding cannabis usage, and the effect this had on how they were perceived in social and professional settings. Users commented that despite conventional medicine having side effects that impacted their ability carry out their jobs they felt forced to be secretive about their cannabis use. Sinclair et al. [30] found that 46.0% of consumers' doctors were aware of their cannabis intake, with 9.3% saying that their doctor had suggested their usage. However, 29.2% of users did not plan on informing their doctor of their cannabis use due to fears of social judgement, with another 29.2% due to their doctor's reaction.

## 4 Discussion

This scoping review of the literature has identified a broad scope of literature around the medicinal use of cannabis by individuals with endometriosis. Considering the burden of endometriosis on an individual and population basis there is a large unmet need. This is highlighted in the population studies contained within the present analysis, where patients are turning to cannabis with or without medical supervision to address symptoms that have not responded to traditional therapies. This review highlighted that several individuals report that cannabis is effective in addressing symptoms of pelvic pain, gastrointestinal discomfort, or co‐morbid psychiatric distress. However, the key finding from this review is that there is a paucity of high‐quality clinical research to sufficiently determine whether medical cannabis is an effective therapy for the symptoms of endometriosis. This is compounded by the heterogeneity of the available literature which examines across different cannabis products, with varying quantities of THC and CBD. Moreover, many in‐human studies include illicitly sourced cannabis which lacks the standardisation of medical cannabis and carries additional risks of contamination and adulteration [31].

The self‐reported data on cannabis efficacy in the management of endometriosis‐associated pain is corroborated by other studies, including systematic reviews of cannabis use in gynaecological pain conditions, including endometriosis, which concluded that cannabis may have a role in the management of chronic non‐cancer pelvic pain [32, 33]. Studies examining the use of cannabis in other conditions causing chronic pain, for example, cancer, fibromyalgia, neuropathic pain, also had similar findings, with varying degrees of certainty [34, 35]. Yet several reviews have reported that there is no high‐quality evidence which can justify the use of cannabinoids in chronic non‐cancer pain [34, 36].

Some randomised trials examining cannabis use in chronic cancer and non‐cancer pain have provided high certainty evidence that cannabis had little to no effect on pain relief or sleep disturbance, whilst increasing nausea and vomiting [36]. However, there are yet to be any RCTs on the use of medicinal cannabis in endometriosis. Two ongoing randomised trials were identified in the literature search. In addition to this, a recent narrative review of CBD and endometriosis highlighted trials in Australia and the United Kingdom that are currently being developed and not yet registered on a clinical trials database [37]. These are eagerly anticipated to help move the field forward. However, there is concern that these are only using isolated formulations of CBD. Previous trials for chronic non‐cancer pain have attempted to repurpose preparations of medical cannabis which have been licensed for other conditions, such as epilepsy and multiple sclerosis‐associated spasticity. The evidence from these trials has been mixed [16, 17, 18]. Considering the challenges with conducting clinical trials and the inherent heterogeneity of medical cannabis products, future RCTs should utilise available observational data presented in this review and pre‐clinical data to choose the most suitable drug candidates.

In those using cannabis for pain management, most used cannabis nearly daily and usually via inhalation or ingestion. Studies on cannabis use in varying forms of chronic pain have also found that smoking or inhaling cannabis is the most common form, followed by oil extracts and edible forms [35]. The evidence is not conclusive, however the evidence included in this review suggests that efficacy increased as THC increased within the THC/CBD ratio. Synthetic cannabis products with high THC/CBD ratios have been associated with moderate improvements in pain severity, though in a study of patient with multiple sclerosis, high THC/CBD ratios were associated with increased risk of study withdrawal due to adverse effects compared to the placebo group [38]. It is important that upcoming trials consider data from the studies included in this review and other sources of real‐world evidence to inform the most important candidates to take forward into phase III trials [39]. Considering the heterogeneity of potential formats and constituent active pharmaceutical ingredients it is important to identify the optimal drug candidate specifically for endometriosis, rather than repurposing preparations that have been licensed for other conditions.

In addition to self‐reported improvements in symptoms, participants in included studies reported reductions in opioids, NSAIDs, anti‐anxiety medications, and non‐opioid analgesics. There is a paucity of evidence to support the use of many of these medications, beyond NSAIDs in addressing the chronic pain associated with endometriosis [40]. It is also an international priority to reduce unnecessary opioid prescribing, due to the known risks of opioid dependence and opioid‐related mortality [41]. A systematic review and network meta‐analysis conducted by Jeddi and colleagues [42] suggested that medical cannabis was associated with a lower risk of discontinuation due to adverse events, when compared to opioids. However, this review did not consider any trials on chronic pain secondary to endometriosis. Observational studies on the use of medical cannabis or the introduction of adult‐use markets have presented mixed evidence of their effects on reducing reliance on opioids. Data from the UK Medical Cannabis Registry published by our group has suggested a reduction in prescribed opioids among patients prescribed medical cannabis for chronic non‐cancer pain [43]. State‐level data examining the effects following changes in cannabis legislation in the United States, however, suggest that there has been no association between increased access to cannabis and reduction in opioid use [44]. There are many confounding factors which may influence opioid prescribing patterns beyond the legalities of cannabis, which may explain why no change was identified. This further underscores the importance of studying the effects of medical cannabis in a controlled trial. Moreover, future trials should consider the impact of medical cannabis on prescribed medications as a secondary outcome measure.

Reports of adverse effects ranged from a minority to around half of respondents. Common symptoms included “feeling high”, and a dry mouth, and some patients ceased using cannabis due to unwanted side effects. However, in general this was less than the side effects experienced with conventional pain medications, and at 52 weeks of follow‐up in patients using 12.5% THC for chronic pain, no difference was seen in adverse events when compared to the control group [39]. Many studies suggest that medical cannabis is relatively well‐tolerated across short‐term follow up [18, 43]. However, there is still concern regarding any potential long‐term effects, the effects of THC on the developing brain and teratogenicity [45]. In a study of Finish women, the mean age of endometriosis diagnosis was 31.6 years [46]. Consequently, further research to understand these risks further are important to determine the potential of medical cannabis as a therapeutic for endometriosis‐associated symptoms.

The major limitations of this review are secondary to the primary literature, which limits the conclusions that can be drawn. Considering the challenges associated with cannabis research, many studies have had to navigate a complex regulatory environment. Consequently, the studies are entirely of an observational nature, without adequate control or randomisation. Therefore it cannot be determined whether the self‐reported improvements in symptoms after consuming cannabis are caused by cannabis itself or another confounding factor. All studies relied on anonymised self‐reports of usage and efficacy of cannabis, leaving them susceptible to multiple biases, including recall, selection, sampling, and nonresponse biases. As these studies were cross‐sectional, follow‐up times were short, and it is difficult to determine the long‐term effects of cannabis, especially as no indications as to the longevity of adverse effects nor the duration of cannabis use were given within the studies. Moreover, they will be subject to recall bias. Finally, there was a large amount of heterogeneity between the studies with respect to formulations and doses used, how endometriosis diagnosis was confirmed and the legalities of cannabis being used. This means direct comparison of the efficacy of cannabis between studies is challenging.

In conclusion, this scoping review found several observational studies which reported that cannabis helped to reduce endometriosis‐associated chronic pain. Most studies included cannabis products which contained a combination of both CBD and THC. The main finding of this review, however, is that there is a paucity of high‐quality prospective longitudinal data, and RCTs to evaluate the safety profile and efficacy of medical cannabis in endometriosis‐associated pain. The two ongoing RCTs identified from ClinicalTrials.gov are therefore eagerly awaited. However, as these are only using isolated CBD as the intervention, there will still be many unanswered questions on the safety and efficacy of medical cannabis as a class of medications. Researchers should consider the observational data contained within this review, where most patients are using cannabis containing both THC and CBD, alongside pre‐clinical data to identify the drug candidates with the highest likelihood of demonstrating efficacy in a trial setting.

## Funding

The authors have nothing to report.

## Conflicts of Interest

Kindha McLaren is a medical student at Imperial College London. He has no shareholdings in pharmaceutical companies. Simon Erridge is a resident doctor and Research Director at Curaleaf Clinic. He is a research fellow at Imperial College London. He has no shareholdings in pharmaceutical companies. Mikael Sodergren is a consultant hepatopancreatobiliary surgeon at Imperial College NHS Trust, London, a senior clinical lecturer at Imperial College London, and the chief medical officer of Curaleaf International. He has no shareholdings in pharmaceutical companies.

## Supporting information


**Table S1:** ajo70081‐sup‐0001‐TableS1.docx.
