# Supplementary material for: A Scoping Systematic Review of Cannabis Use in Endometriosis
Source: Aust N Z J Obstet Gynaecol. 2025 Dec 9;66(1):e70081. doi: 10.1111/ajo.70081 (PMC12920050; doi:10.1111/ajo.70081)
Supplement: Supplementary file 1 — Table S1: ajo70081‐sup‐0001‐TableS1.docx. [file AJO-66-0-s001.docx]

**Additional file 1**

**Search strategies and results**

**Table S1: Summary of Databases Searched**

| **Table** | **Vendor/ Interface** | **Database** | **Date searched** | **Database update** | **Searcher(s)** |
| --- | --- | --- | --- | --- | --- |
| 1a | Ovid | MEDLINE | 16/01/2024 | 1946 to January 12 2024 | S. Erridge; K. McLaren |
| 1b | National Library of Medicine | PubMed | 16/01/2024 | January 15 2024 | S. Erridge; K. McLaren |
| 1c | Ovid | EMBASE | 16/01/2024 | 1947 to January 12 2024 | S. Erridge; K. McLaren |

**Table S1a: Ovid MEDLINE search strategy**

| **Provider/Interface** | Ovid |
| --- | --- |
| **Database** | MEDLINE |
| **Date searched** | 16/01/2024 |
| **Database update** | 1946 to January 12 2024 |
| **Search developer(s)** | S. Erridge; K. McLaren |
| **Limit to English** | No |
| **Date Range** | 1946-2024 |

| **#** | **Query** |
| --- | --- |
| 1 | Endometriosis.af. |
| 2 | Endometrio$.af. |
| 3 | 1 or 2 |
| 4 | Cannabis.af. |
| 5 | (cannabis or hemp or marijuana or ganja or hashish or marihuana or bhang or cannabinoid*).af. |
| 6 | (dronabinol or marinol or nabilone or cesamet or "HU 211" or dexanabinol or nabiximols or sativex or dronabinol or tetrahydrocannabinol).af. |
| 7 | (cannabidiol or epidiolex or epidyolex).af. |
| 8 | cannabinol.af. |
| 9 | 4 or 5 or 6 or 7 or 8 |
| 10 | 3 and 9 |

**S1b: PubMed search strategy**

| **Provider/Interface** | National Library of Medicine |  |
| --- | --- | --- |
| **Database** | PubMed | |
| **Date searched** | 16/01/2024 | |
| **Database update** | January 15 2024 | |
| **Search developer(s)** | S. Erridge; K. McLaren | |
| **Limit to English** | No | |
| **Date Range** | -15/01/2024 | |

| **#** | **Query** |
| --- | --- |
| 1 | Endometriosis.af. |
| 2 | Endometrio*.af. |
| 3 | 1 or 2 |
| 4 | Cannabis.af. |
| 5 | (cannabis or hemp or marijuana or ganja or hashish or marihuana or bhang or cannabinoid*).af. |
| 6 | (dronabinol or marinol or nabilone or cesamet or "HU 211" or dexanabinol or nabiximols or sativex or dronabinol or tetrahydrocannabinol).af. |
| 7 | (cannabidiol or epidiolex or epidyolex).af. |
| 8 | cannabinol.af. |
| 9 | 4 or 5 or 6 or 7 or 8 |
| 10 | 3 and 9 |

**S1c: Ovid EMBASE search strategy**

| **Provider/Interface** | Ovid |  |
| --- | --- | --- |
| **Database** | EMBASE | |
| **Date searched** | 16/01/2024 | |
| **Database update** | 1946 to January 12 2024 | |
| **Search developer(s)** | S. Erridge; K. McLaren | |
| **Limit to English** | No | |
| **Date Range** | 1946-2024 | |

| **#** | **Query** |
| --- | --- |
| 1 | Endometriosis.af. |
| 2 | Endometrio$.af. |
| 3 | 1 or 2 |
| 4 | Cannabis.af. |
| 5 | (cannabis or hemp or marijuana or ganja or hashish or marihuana or bhang or cannabinoid*).af. |
| 6 | (dronabinol or marinol or nabilone or cesamet or "HU 211" or dexanabinol or nabiximols or sativex or dronabinol or tetrahydrocannabinol).af. |
| 7 | (cannabidiol or epidiolex or epidyolex).af. |
| 8 | cannabinol.af. |
| 9 | 4 or 5 or 6 or 7 or 8 |
| 10 | 3 and 9 |
